# Unusual acute lupus hemophagocytic syndrome – a test of diagnostic criteria: a case report

**DOI:** 10.1186/s13256-017-1339-7

**Published:** 2017-07-07

**Authors:** Wijetunga Mudalige Udai Akalanka Wijetunga, Ravindra Laxman Satarasinghe, Balasuriya Mudiyanselage Dayananda, Ganhewage Kokila Darshani

**Affiliations:** 1 Postgraduate Institute of Medicine, University of Colombo, Colombo, Sri Lanka; 2 Sri Jayawardenapura General Hospital, Thalapathpitiya, Nugegoda Sri Lanka

**Keywords:** Systemic lupus erythematosus, Acute lupus hemophagocytic syndrome, Hemophagocytic lymphohistiocytosis, Case report

## Abstract

**Background:**

Hemophagocytic lymphohistiocytosis is an aggressive life-threatening syndrome of excessive immune activation. Hemophagocytic lymphohistiocytosis due to systemic lupus erythematosus is described as acute lupus hemophagocytic syndrome. Acute lupus hemophagocytic syndrome presenting with negative antinuclear antibody is uncommon.

**Case presentation:**

A 57-year-old Sri Lankan woman presented with intermittent fever, weight loss, episodic confusion, and alopecia for 3 months. Investigations showed pancytopenia. Her erythrocyte sedimentation rate was 76 mm/hour and C-reactive protein was 2 mg/l. Septic screen was negative except for a positive sputum culture for Gram-negative coliforms. Chest X-ray was normal. Direct antiglobulin test was positive. Fever persisted with clinical worsening despite treatment with intravenous antibiotics. Antinuclear antibodies and double-stranded-deoxyribonucleic acid antibodies were negative. Bone marrow aspiration revealed features compatible with hemophagocytosis. Her serum ferritin and triglycerides were elevated. Diagnosis of hemophagocytic lymphohistiocytosis due to an evolving autoimmune disorder was made and she was treated with steroids. She showed a dramatic improvement and was discharged on oral steroids.

After 6 months, while the steroids were being tapered she experienced oral ulcers, frothy urine, and ankle swelling and she was rehospitalized. Urine analysis revealed proteinuria with active sediment. Antinuclear antibodies and double-stranded-deoxyribonucleic acid antibodies were positive. Complement C3 and C4 were reduced. A renal biopsy revealed class IV-G lupus nephritis with immunofluorescence pattern consistent with systemic lupus erythematosus. Steroid dose was increased and mycophenolate mofetil was commenced. She improved.

**Conclusions:**

This case showcases an uncommon presentation of acute lupus hemophagocytic syndrome with initial negative antinuclear antibody probably due to its cytokine-mediated pathogenesis. This is the first such reported case in South Asia to the best of our knowledge. According to the American College of Rheumatology criteria, our patient did not fulfill the criteria for systemic lupus erythematosus diagnosis for the initial hospitalization. But, according to the 2012 Systemic Lupus International Collaborating Clinics criteria, she did fulfill the criteria for systemic lupus erythematosus even in the first hospitalization which was subsequently proven with renal biopsy findings. This case confirms the increased sensitivity of Systemic Lupus International Collaborating Clinics criteria over American College of Rheumatology criteria in diagnosis of systemic lupus erythematosus.

## Background

Hemophagocytic lymphohistiocytosis (HLH) is an aggressive and life-threatening syndrome of excessive immune activation characterized by increased proliferation and activation of benign macrophages with hemophagocytosis in the bone marrow and other reticuloendothelial systems [1, 2]. HLH is also known to cause proliferation and activation of T lymphocytes and macrophages producing an excessive inflammatory response and hypersecretion of cytokines [3]. HLH is a rare disease in adults and the incidence is one patient per million persons per year [1]. HLH can occur as a familial primary form or secondary to infections, rheumatologic diseases, medications, and neoplasms [3]. Reactive hemophagocytosis occurring due to underlying active systemic lupus erythematosus (SLE) is described as acute lupus hemophagocytic syndrome (ALHS) [4].

ALHS is usually associated with high titers of antinuclear antibodies (ANAs) and hypocomplementemia, suggesting immune complex-mediated mechanisms in the pathogenesis of ALHS [5]. The incidence of autoimmune-associated hemophagocytic syndrome in SLE was found to be 4.6% in one study [6].

The usual patient presentation of HLH is characterized by prolonged fever, weight loss, hepatosplenomegaly, lymphadenopathy, coagulopathy, pancytopenia, liver function abnormalities, hyperferritinemia, and hypertriglyceridemia [2, 3]. It is sometimes difficult to differentiate an acute SLE case from HLH due to them having many common features. To make matters worse, it is not easy to differentiate hemophagocytic syndromes occurring secondary to infection from those occurring due to a rheumatological cause like SLE. It is essential that this distinction is made because HLH due to SLE responds rapidly to steroids and immunosuppressants [1].

## Case presentation

A 57-year-old previously healthy Sri Lankan woman presented to our hospital with 3 months’ history of intermittent fever, anorexia, and weight loss. She had lost 8 kg over this period. A worsening of the fever characterized by daily episodes with spikes prompted admission. She complained of significant hair loss and on and off confusional episodes over the last 2 weeks, but denied symptoms of focal infections including respiratory symptoms. Also, she disaffirmed any history of arthritis and features of autoimmune diseases such as photosensitive rashes, oral ulcers, or symptoms of Raynaud’s phenomenon. On examination her body mass index was 16 kg/m^2^. She was febrile with a temperature of 37.8 °C (100 °F), pale, and non-scarring alopecia was evident. Her other vital parameters were normal (blood pressure 130/80 mmHg, pulse rate 92/minute, respiratory rate 16 breaths/minute). The rest of the examination was unremarkable.

A full blood count done a month before admission revealed a bicytopenia with leukopenia and thrombocytopenia. Her erythrocyte sedimentation rate (ESR) was 70 mm/hour and C-reactive protein (CRP) was 6 mg/l at that time. The differential diagnoses considered at this point were infections such as tuberculosis (TB), infective endocarditis, hematological and solid organ malignancies, and autoimmune diseases.

Initial full blood count after admission showed a pancytopenia which was confirmed on blood picture: white blood cell count (WBC) 1690/mm^3^, hemoglobin (Hb) 6.0 g/dl, and platelets 102,000/mm^3^. Her ESR was 76 mm/hour and CRP was 2 mg/l. A sputum culture was positive for Gram-negative coliforms. A chest X-ray was normal. Intravenously administered antibiotics were started according to the antibiotic sensitivity tests (ABSTs) but her fever continued. Repeated blood and urine cultures were negative. A urine full report was normal. A diagnosis of a respiratory tract infection was unlikely because she did not have any respiratory tract symptoms, examination of her lungs was normal, her CRP was normal repeatedly, and her fever did not subside despite continuing apparently sensitive intravenously administered antibiotics with no clinical improvement.

**Fig. 1:**
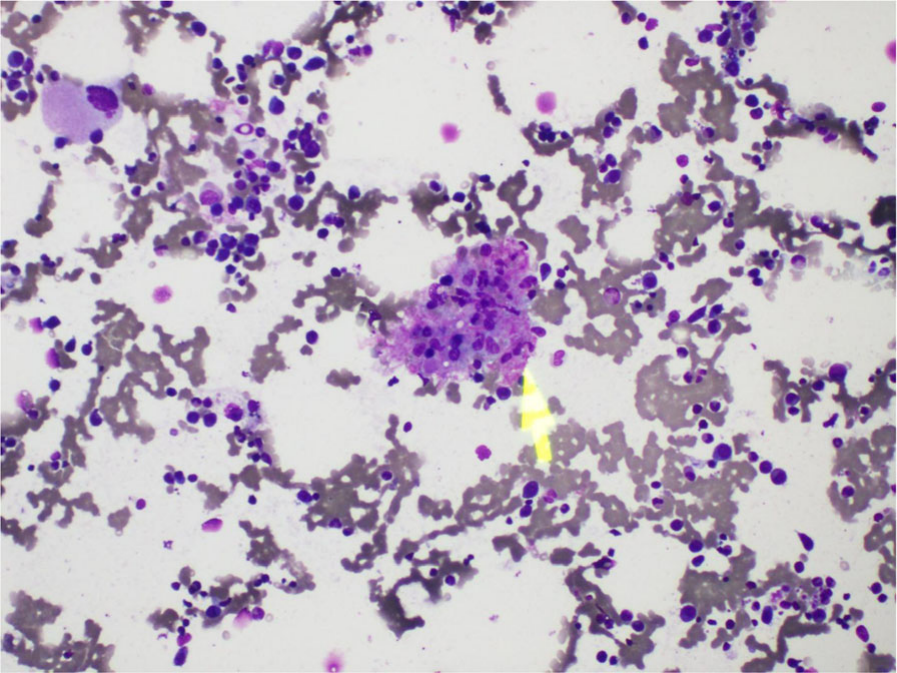
Bone marrow biopsy (x20 magnification) : Phagocytosis of RBC and WBC are seen (*arrow*)

**Fig. 2:**
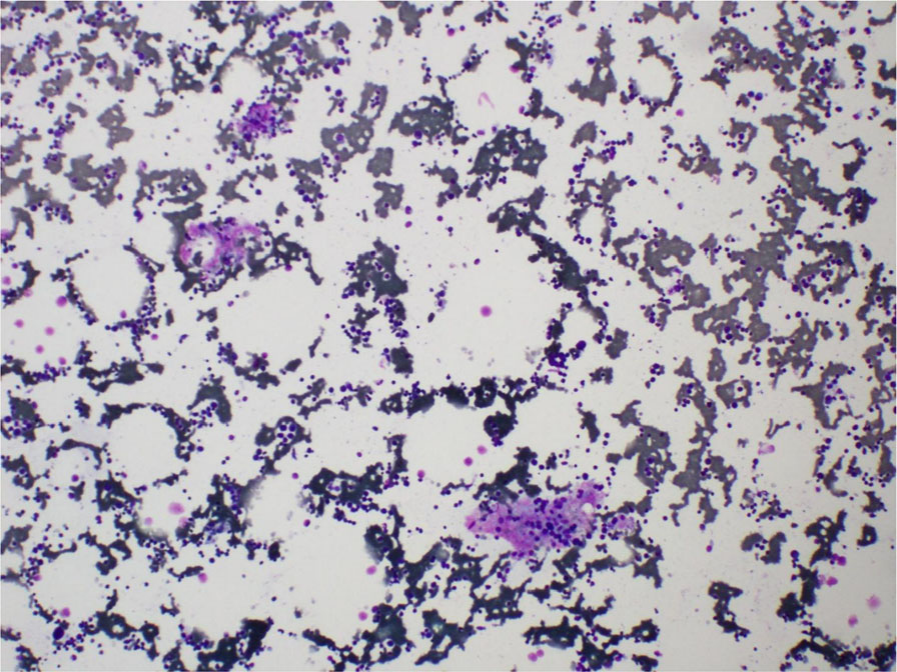
Bone marrow biopsy (x4 magnification) : Multiple phagocytic foci are seen

**Fig. 3:**
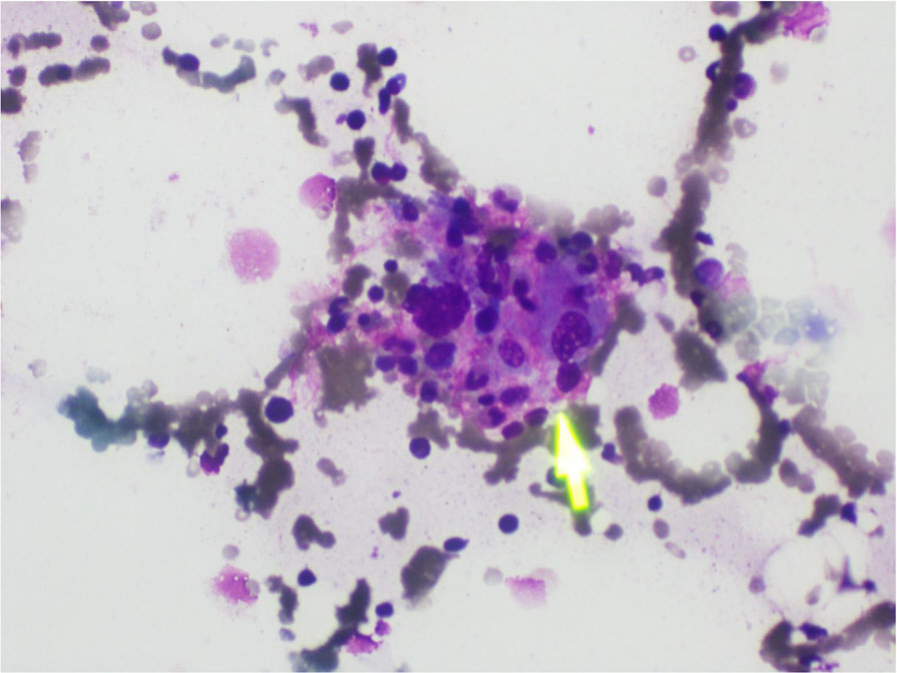
Bone marrow biopsy (x40 magnification) : Phagocytic focus (*arrow*) seen under high power magnification

**Fig. 4:**
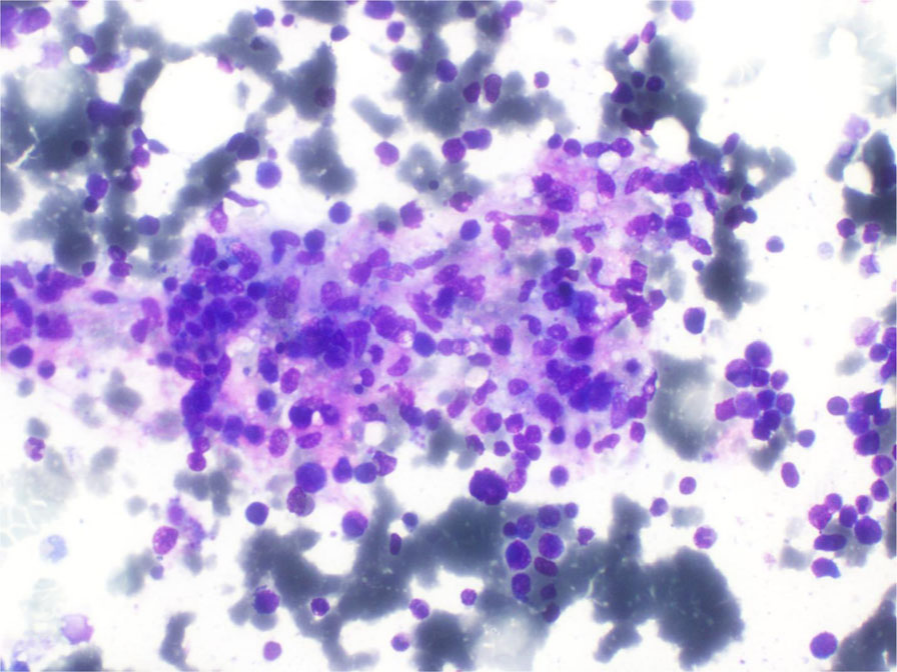
Bone marrow biopsy (x40 magnification) : Phagocytic focus with increased number of phagocytosed cells seen under high power magnification

A two-dimensional echocardiogram revealed no evidence of infective endocarditis. Sputum for acid-fast bacilli and Mantoux test were negative. Bone marrow aspiration was done and it revealed hemophagocytic activity in her bone marrow suggestive of primary or secondary hemophagocytic syndrome. There were no features of hematological malignancy in the bone marrow (Figs. 1, 2, 3 and 4). Serum ferritin was highly elevated (>1650 ng/dl). Serum lactate dehydrogenase (LDH) was elevated (926 U/l). Serum triglyceride level was 270 mg/dl. Serum protein electrophoresis showed only a polyclonal gammopathy, thus excluding the presence of a monoclonal band. Carcinoembryonic antigen (CEA) level was normal.

Epstein–Barr virus (EBV), cytomegalovirus (CMV), and rubella virus antibodies were negative. Screening for hepatitis B, hepatitis C, and human immunodeficiency virus (HIV) was negative. Bone marrow for TB polymerase chain reaction (PCR) and culture were negative. An ultrasound scan of her abdomen and pelvis was normal.

An autoimmune screen was negative with ANA, anti-double-stranded-deoxyribonucleic acid (dsDNA), rheumatoid factor, cytoplasmic antineutrophil cytoplasmic antibody (c-ANCA), and perinuclear antineutrophil cytoplasmic antibody (p-ANCA) being negative. A direct antiglobulin test was positive with specificity to immunoglobulin A (IgA).

Due to her persistent fever, a diagnosis of hemophagocytic syndrome secondary to an evolving autoimmune disease was made and she was started on intravenously administered methylprednisolone pulses. She was given intravenously administered methylprednisolone 1 g daily for 3 days which was followed by orally administered prednisolone 60 mg daily. She showed a dramatic response with clinical improvement, resolution of fever, resolution of pancytopenia, reduction in LDH and ferritin, and reduction in ESR. She was discharged and was sent with a tapering dose of orally administered prednisolone. On follow-up, she reported no adverse effects of medication and she was compliant with her treatment regime.

After 6 months while on a tapering dose of prednisolone, she experienced intermittent fever, oral ulcers, frothy urine, and ankle swelling for 3 weeks which motivated a second admission. An evaluation revealed subnephrotic range proteinuria with active sediment on urine analysis. ANA (>1/80) and anti-dsDNA (>1/10) were positive. Complement C3 and C4 (74 mg/dl and 2.9 mg/dl) were reduced. A renal biopsy was done and it revealed class IV-G lupus nephritis with immunofluorescence pattern consistent with SLE. A diagnosis of SLE with renal involvement was made. A summary of investigation results are given in Table 1.

**Table 1:** Investigation summary

	First admission	Second admission
White blood cells	1690/mm^3^	4900/mm^3^
Lymphocyte count	740/mm^3^	420/mm^3^
Neutrophil count	910/mm^3^	4210/mm^3^
Hemoglobin	6.0 g/dl	8.4 g/dl
Platelets	102,000/mm^3^	260,000/mm^3^
ESR	76 mm/hour	120 mm/hour
CRP	2 mg/dl	<6 mg/dl
LDH	926 u/l	482 u/l
Ferritin	>1650 ng/ml	>1650 ng/ml
AST	106 u/l	105 u/l
ALT	64 u/l	97 u/l
ALP	85 iu/l	166 iu/l
Serum bilirubin	0.4 mg/dl	0.3 mg/dl
Serum protein	5.4 g/dl	6.4 g/dl
Serum albumin	3.0 g/dl	3.8 g/dl
Serum sodium	131 mEq/l	140 mEq/l
Serum potassium	4.6 mEq/l	4.1 mEq/l
Serum creatinine	89 micromol/l	88 micromol/l

*ALP* alkaline phosphatase, *ALT* alanine aminotransferase, *AST* aspartate aminotransferase, *CRP* C-reactive protein, *ESR* erythrocyte sedimentation rate, *LDH* lactate dehydrogenase

**Fig. 5:**
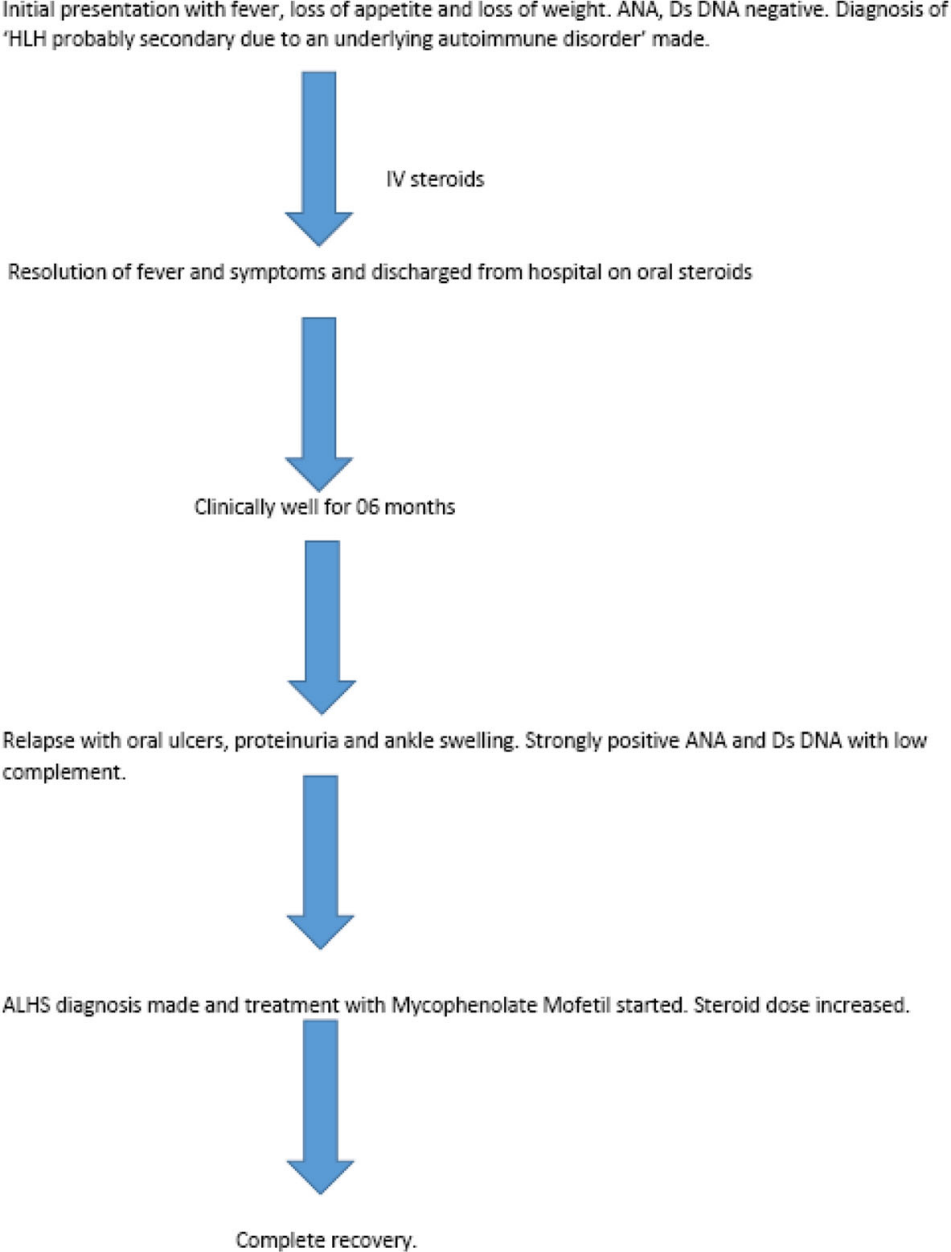
*ALHS* acute lupus hemophagocytic syndrome, *ANA* antinuclear antibody, *Ds DNA* double-stranded-deoxyribonucleic acid, *HLH* hemophagocytic lymphohistiocytosis, *IV* intravenous

She was started on orally administered prednisolone 60 mg daily and mycophenolate mofetil with good response. On follow-up she was clinically improved and has remained asymptomatic to date (9 months after discharge from hospital) while on immunosuppressive treatment. The prednisolone dose was gradually reduced and maintained at a dose of 7.5 mg daily. Mycophenolate mofetil was continued. Thus with this new irrevocable evidence of SLE, the diagnosis of the previous admission was revised as ALHS (Fig. 5).

## Discussion

An updated set of diagnostic criteria for HLH was proposed in 2004 by The Histiocyte Society. The diagnosis needs either a molecular diagnosis consistent with HLH including the identification of pathologic mutations of *PRF1*, *UNC13D*, or *STX11* or the presence of at least five out of eight features: fever, splenomegaly, bi/pancytopenia, hypertriglyceridemia or hypofibrinogenemia, ferritin >500 ng/ml, hemophagocytosis in the bone marrow/lymph nodes/spleen, low natural killer cell activity, soluble CD25 (soluble interleukin-2 receptor) >2400 U/ml [7, 8].

This patient’s initial presentation fulfilled the criteria for HLH according to the above criteria although the underlying cause was not clear at the time. The combination of suggestive bone marrow results, elevated serum triglycerides and ferritin, and fever and pancytopenia completed the diagnostic criteria for HLH in this patient.

The main underlying causes for HLH are autoimmunity, infection, and malignancy [9]. The signs of an evolving autoimmune disease were evident even at the initial presentation. They included persistent fever and symptoms despite treating with intravenously administered antibiotics, presence of significant alopecia, direct antiglobulin test being positive and rapid improvement after treatment with steroids. She did not test positive for ANA or dsDNA tests initially and did not fulfill the diagnostic criteria for SLE according to the 1982 American College of Rheumatology (ACR) criteria or its 1997 update. Thus the diagnosis was initially given as HLH probably due to an underlying evolving autoimmune disease.

Of interest, according to the 1982 ACR criteria and even its 1997 update, our patient did not fulfil the criteria for SLE diagnosis for the initial hospitalization. These two classifications need at least four out of 11 criteria to be present for a diagnosis of SLE to be made. But this patient only fulfilled two criteria at best. According to the 2012 Systemic Lupus International Collaborating Clinics (SLICC) revised ACR SLE classification criteria [10], our patient fulfilled the criteria for SLE even in the first hospitalization. She had four of the diagnostic criteria including non-scarring alopecia, leukopenia/lymphopenia, history of acute confusional states, and positive direct antiglobulin test. A fifth criterion was almost fulfilled with a platelet count of 102,000/mm^3^ being just above 100,000/mm^3^. Thus the diagnosis of ALHS is further confirmed.

Several days after our patient’s admission, it was clear to us that her fever was not due to an infection although the sputum culture turned out positive for coliforms. Coliforms are well recognized to be isolated in sputum cultures in hospitalized patients where they colonize the upper respiratory tracts, especially in patients receiving long-term antibiotics as in the case of our patient. In the absence of other signs of infection coliforms isolates should be regarded as saprophytes in such clinical scenarios [11–13]. The likelihood of isolating an upper respiratory tract-colonizing organism is further increased in this patient especially because of the absence of a productive cough. Thus, she may have given a suboptimal specimen mostly consisting of saliva and upper respiratory tract secretions, leading to isolation of a coliform growth in the sputum culture. It was further confirmed when treatment with sensitive antibiotics failed to resolve her fever. Repeatedly sterile blood cultures, persistently normal CRP, absence of any respiratory symptoms or signs, and normal chest X-rays made the diagnosis of a respiratory tract infection unlikely.

Although the possibility of an infection is low in this patient, infections are known to trigger the onset of SLE [14, 15]. Infections are responsible for significant morbidity and mortality in patients with SLE [16]. Patients with SLE have been found to be susceptible to pneumonia, urinary tract infections, and bacteremia without an identified focus [17]. This could be due to intrinsically associated immune deficiency or due to drugs used in the treatment of SLE [18].

Malignancies associated with HLH include malignant lymphoma, leukemias, and Waldenström’s macroglobulinemia [9]. These were considered very unlikely due to unsuggestive bone marrow examination and blood picture. These diagnoses were further excluded by a normal chest X-ray and ultrasound scan of her abdomen and by exclusion of a monoclonal band in the serum protein electrophoresis.

The occurrence of ALHS is usually seen in the setting of high ANA levels and hypocomplementemia indicating immune complex-mediated mechanisms in its pathogenesis [5]. But this patient had negative ANA in the initial presentation leading to masking of the underlying disease. Similar cases of ALHS with low titers of ANA have been reported and those authors have proposed a cytokine-mediated activation of histiocytes rather than an immune complex-mediated process [5]. Thus it is most likely that our patient’s initial disease presentation with HLH with a negative ANA was generated by cytokine-mediated mechanisms.

Most reported cases of ALHS have had positive and mostly high titers of ANA on presentation, but our patient interestingly presented with a negative ANA test and did not satisfy the diagnostic criteria for SLE according to the 1982 ACR criteria or its 1997 update, although she did fulfill the criteria for SLE when the 2012 SLICC revised ACR SLE classification criteria were considered. Her dramatic and persistent response to steroids strongly suggested an autoimmune etiology even when autoimmune markers were negative.

During her subsequent hospitalization, the evolving autoimmunity further revealed itself with strongly positive ANA and anti-dsDNA antibodies and the diagnosis of SLE was confirmed with positive renal biopsy findings. In retrospect, her autoimmunity must have responded to the steroids initially, but when the dose was reduced it flared up and it revealed itself with strongly positive ANA and dsDNA. Thus this case highlights an unusual presentation of ALHS with diagnostic difficulty probably due to its cytokine-mediated underlying pathogenesis. Due to its unusual presentation, the diagnosis of ALHS could only be made in retrospect with the evolution of the disease process.

## Conclusions

This case is interesting for many reasons. First, the initial presentation of ALHS with negative ANA and dsDNA is uncommon. Second, the initial presentation fulfils the diagnostic criteria for SLE according to the 2012 SLICC revised ACR SLE classification criteria but not with the 1982 ACR criteria or its 1997 update. Thus this case further indicates the sensitivity of the 2012 SLICC revised ACR SLE classification criteria. Third, this is the first such unusual case of ALHS reported in Sri Lanka and South Asia to the best of our knowledge.

Thus this case highlights the importance of having a high index of suspicion and the need to have close follow-up to detect ALHS when a patient presents with a picture of HLH, even when autoimmune markers are negative and the patient does not fulfill all the criteria for the diagnosis of SLE initially.

## Abbreviations

ABST: Antibiotic sensitivity test
ACR: American College of Rheumatology
ALHS: Acute lupus hemophagocytic syndrome
ANA: Antinuclear antibody
c-ANCA: Cytoplasmic antineutrophil cytoplasmic antibody
CEA: Carcinoembryonic antigen
CMV: Cytomegalovirus
CRP: C-reactive protein
dsDNA: Double-stranded-deoxyribonucleic acid
EBV: Epstein–Barr virus
ESR: Erythrocyte sedimentation rate
Hb: Hemoglobin
HLH: Hemophagocytic lymphohistiocytosis
IgA: Immunoglobulin A
LDH: Lactate dehydrogenase
p-ANCA: Perinuclear antineutrophil cytoplasmic antibody
PCR: Polymerase chain reaction
SLE: Systemic lupus erythematosus
SLICC: Systemic Lupus International Collaborating Clinics
TB: Tuberculosis
WBC: White blood cell count
